# Repeat Ivermectin Mass Drug Administrations for Malaria Control II: Protocol for a Double-blind, Cluster-Randomized, Placebo-Controlled Trial for the Integrated Control of Malaria

**DOI:** 10.2196/41197

**Published:** 2023-03-20

**Authors:** Brian D Foy, Anthony Some, Tereza Magalhaes, Lyndsey Gray, Sangeeta Rao, Emmanuel Sougue, Conner L Jackson, John Kittelson, Hannah C Slater, Teun Bousema, Ollo Da, A Gafar V Coulidiaty, McKenzie Colt, Martina Wade, Kacey Richards, A Fabrice Some, Roch K Dabire, Sunil Parikh

**Affiliations:** 1 Center for Vector-Borne Infectious Diseases Department of Microbiology, Immunology and Pathology Colorado State University Fort Collins, CO United States; 2 Institut de Recherche en Sciences de la Santé Direction Régionale de l’Ouest Bobo-Dioulasso Burkina Faso; 3 Department of Entomology Texas A&M University College Station, TX United States; 4 Department of Preventive and Social Medicine School of Medicine Universidade Federal da Bahia Salvador Brazil; 5 Department of Clinical Sciences Colorado State University Fort Collins, CO United States; 6 Department of Biostatistics and Informatics University of Colorado School of Public Health Aurora, CO United States; 7 Malaria and Neglected Tropical Diseases Program for Appropriate Technology in Health Seattle, WA United States; 8 Radboud Institute for Health Sciences Radboud University Medical Center Nijmegen Netherlands; 9 Department of Epidemiology of Microbial Diseases Yale School of Public Health New Haven, CT United States

**Keywords:** malaria, ivermectin, mass drug administration, mosquito, Anopheles, endectocide, cluster-randomized, clinical trial, seasonal malaria chemoprevention, transmission

## Abstract

**Background:**

The gains made against malaria have stagnated since 2015, threatened further by increasing resistance to insecticides and antimalarials. Improvement in malaria control necessitates a multipronged strategy, which includes the development of novel tools. One such tool is mass drug administration (MDA) with endectocides, primarily ivermectin, which has shown promise in reducing malaria transmission through lethal and sublethal impacts on the mosquito vector.

**Objective:**

The primary objective of the study is to assess the impact of repeated ivermectin MDA on malaria incidence in children aged ≤10 years.

**Methods:**

Repeat Ivermectin MDA for Malaria Control II is a double-blind, placebo-controlled, cluster-randomized, and parallel-group trial conducted in a setting with intense seasonal malaria transmission in Southwest Burkina Faso. The study included 14 discrete villages: 7 (50%) randomized to receive standard measures (seasonal malaria chemoprevention [SMC] and bed net use for children aged 3 to 59 months) and placebo, and 7 (50%) randomized to receive standard measures and monthly ivermectin MDA at 300 μg/kg for 3 consecutive days, provided under supervision to all eligible village inhabitants, over 2 successive rainy seasons. Nonpregnant individuals >90 cm in height were eligible for ivermectin MDA, and cotreatment with ivermectin and SMC was not permitted. The primary outcome is malaria incidence in children aged ≤10 years, as assessed by active case surveillance. The secondary safety outcome of repeated ivermectin MDA was assessed through active and passive adverse event monitoring.

**Results:**

The trial intervention was conducted from July to November in 2019 and 2020, with additional sampling of humans and mosquitoes occurring through February 2022 to assess postintervention changes in transmission patterns. Additional human and entomological assessments were performed over the 2 years in a subset of households from 6 cross-sectional villages. A subset of individuals underwent additional sampling in 2020 to characterize ivermectin pharmacokinetics and pharmacodynamics. Analysis and unblinding will commence once the database has been completed, cleaned, and locked.

**Conclusions:**

Our trial represents the first study to directly assess the impact of a novel approach for malaria control, ivermectin MDA as a mosquitocidal agent, layered into existing standard-of-care interventions. The study was designed to leverage the current SMC deployment infrastructure and will provide evidence regarding the additional benefit of ivermectin MDA in reducing malaria incidence in children.

**Trial Registrations:**

ClinicalTrials.gov NCT03967054; https://clinicaltrials.gov/ct2/show/NCT03967054 and Pan African Clinical Trials Registry PACT201907479787308; https://pactr.samrc.ac.za/TrialDisplay.aspx?TrialID=8219

**International Registered Report Identifier (IRRID):**

DERR1-10.2196/41197

## Introduction

### Background and Previous Studies

Widespread implementation of new malaria control tools since the turn of the century has dramatically reduced malaria parasite transmission and malaria-related morbidity and mortality across the globe, especially in African countries. Improved access to vector control interventions (indoor residual spraying [IRS] and insecticide-treated bed nets [ITNs]), point-of-care diagnostics (rapid diagnostic tests [RDTs]), and efficacious treatment (artemisinin-based combination therapies) have all contributed to this success. ITNs appear to have made the most dramatic impact, with models estimating that 68% of the decline in *Plasmodium falciparum* prevalence in Africa between 2000 and 2015 could be attributed to this vector-focused intervention [1]. In addition to these tools, the use of intermittent preventive treatment in pregnancy (IPTp) and seasonal malaria chemoprevention (SMC) have contributed to morbidity reductions. IPTp entails the administration of at least 3 antenatal doses of sulfadoxine-pyrimethamine (SP) to pregnant women living in malaria endemic regions. SMC, implemented primarily in the seasonal Sahel region of West Africa, entails the use of amodiaquine (AQ) and SP in children aged 3 to 59 months, administered over 3 days during each month of the rainy season [2]. Studies suggest that SMC is very effective in reducing malaria morbidity, with reductions in incidence of up to 70% [2]; the target age group was recently widened to include all age groups at risk of severe disease, and several countries are already piloting or implementing SMC in all children aged <10 years [2]. In most settings, Ministries of Health (MoHs) work with local communities to deliver SMC door to door over 4 months, even though other delivery approaches and tailoring of the number of months of administration are being discussed and implemented in different regions [2].

Unfortunately, despite these impactful interventions, gains against malaria have been uneven across Africa and have largely stagnated or reversed since 2015 [3]. Given the major contribution of vector control interventions to malaria control, it is not surprising that widespread mosquito resistance to the insecticide classes being used in ITNs and IRS (including pyrethroids, carbamates, and organophosphates) and behavioral avoidance (biting outdoors, resting outdoors, or biting at times when individuals are not sleeping under ITNs) are principal reasons for this stagnation in gains. For example, despite high SMC and ITN coverage, Burkina Faso remains as one of the countries with the highest malaria burden, as all major malaria vectors are resistant to pyrethroids [4].

In light of these challenges, it is clear that additional control methods are needed against the *Anopheles* vector. A promising option is the use of endectocides, drugs that kill both endoparasites (such as parasitic worms) and ectoparasites (such as fleas, lice, and mosquitoes) that feed on treated hosts [5-8]. The most well studied of these is ivermectin, a semisynthetic avermectin derivative that was first licensed in 1981 and has been administered for decades at a dose of 150 to 400 μg/kg once or twice a year as mass drug administrations (MDAs) to treat and eliminate onchocerciasis, lymphatic filariasis, and scabies [9]. Importantly, it has a broad spectrum of activity that also targets invertebrate-specific ligand-gated ion channels, hyperpolarizing their neurons and causing flaccid paralysis, death, and other sublethal effects [10]. Our team and others have pioneered the development of ivermectin as a novel malaria transmission control tool through laboratory and natural field experiments [11-14] and recent clinical trials [15-17], including a recent cluster-randomized trial (Repeat Ivermectin MDA for Malaria Control [RIMDAMAL]; conducted by our group) of repeated low-dose (150 μg/kg) ivermectin MDA during the rainy season in Burkina Faso that reduced malaria incidence by 20% in children aged <5 years [18].

### Goal of This Study

Although RIMDAMAL supported the potential impact of repeated MDA with ivermectin on malaria incidence, the doses and frequency were not optimized for either maximizing efficacy or ease of implementation. In particular, low-dose ivermectin is unlikely to achieve sufficient and sustained drug levels to reduce malaria transmission if given monthly. On the basis of pharmacokinetic and pharmacodynamic modeling, we determined that a repeated monthly dose of 300 μg/kg daily for 3 days would be expected to achieve sufficient blood levels to have a clinically relevant impact on malaria rates in children [1,7,19]. In addition, to facilitate the implementation of repeated ivermectin MDA on a wide scale, we hypothesized that combining the delivery of SMC (already standard practice in most of Sahel) with ivermectin would facilitate the deployment of our intervention and provide a multifaceted and layered approach for malaria control, targeting both the parasite and the mosquito simultaneously, which is critical to making further gains toward reducing the burden of malaria [1,7,20]. Therefore, we designed the RIMDAMAL II trial, a double-blind, cluster-randomized, and placebo-controlled trial for integrated control of malaria. The trial aims to assess the impact of a combined approach of repeated ivermectin MDA and SMC on malaria incidence in children compared with that of repeated placebo MDA and SMC. The trial was implemented in the high-transmission, highly seasonal Diébougou region of Southwest Burkina Faso, the same general location as RIMDAMAL.

## Methods

### Study Design Overview

**Figure 1:**
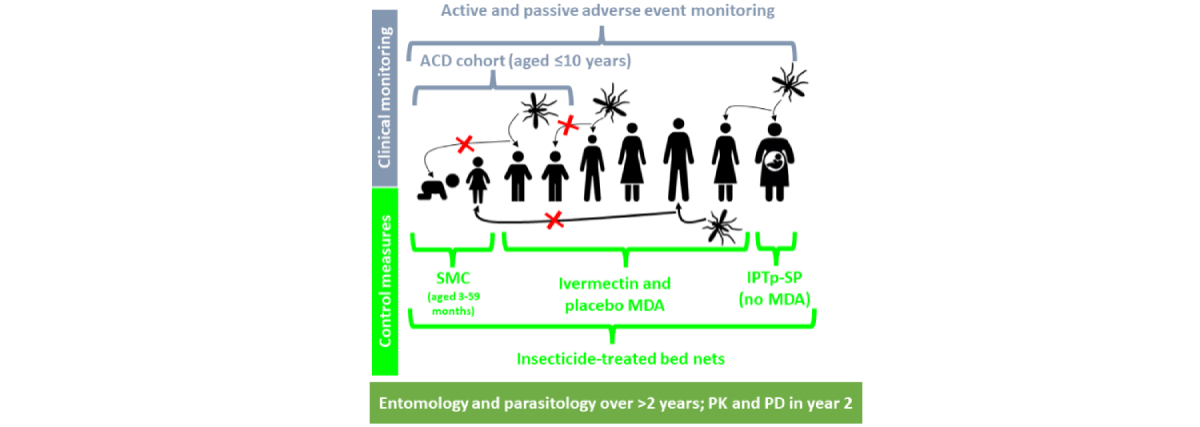
Overall study design showing which groups received each malaria control intervention and which age demographic constituted the active case detection (ACD) cohort [1]. IPTp: intermittent preventive treatment in pregnancy; MDA: mass drug administration; PD: pharmacodynamics; PK: pharmacokinetics; SMC: seasonal malaria chemoprevention; SP: sulfadoxine-pyrimethamine.

**Figure 2:**
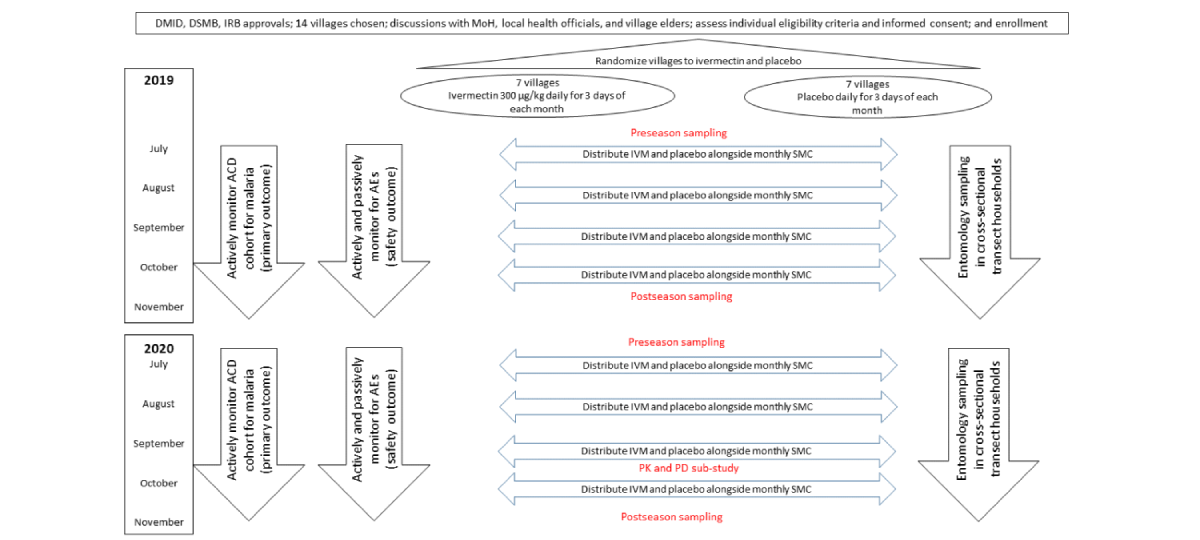
Diagram of study interventions and follow-up for primary and secondary outcomes. ACD: active case detection; AE: adverse event; DMID: Division of Microbiology and Infectious Diseases; DSMB: Data Safety Monitoring Board; IRB: institutional review board; IVM: ivermectin; MoH: Ministry of Health; PD: pharmacodynamics; PK: pharmacokinetics; SMC: seasonal malaria chemoprevention.

RIMDAMAL II is a double-blind, placebo-controlled, cluster-randomized, and parallel-group trial (ClinicalTrials.gov: NCT03967054; pactr.org: PACT201907479787308) designed to determine the efficacy of adding seasonal ivermectin MDA to the standard-policy malaria control measures in Sahel (SMC in children aged 3 to 59 months, long-lasting ITN coverage, and IPTp), for reducing the incidence of uncomplicated malaria episodes among children in enrolled villages (Figure 1). The study includes 14 randomized villages (n=7, 50% control and n=7, 50% intervention clusters). The primary end point is the incidence of malaria over 2 consecutive ivermectin MDA intervention seasons in children aged ≤10 years (Figure 2).

### Study Objectives

#### Primary Objective

*Primary objective* is to assess the impact of a combined approach of repeated ivermectin MDA and SMC on malaria incidence in children aged ≤10 years (active case detection [ACD] cohort; assessed weekly), with the purpose of testing the superiority of ivermectin compared with placebo for reducing malaria incidence. Our hypothesis is that ivermectin MDAs (the intervention), coinciding with standard-of-care control procedures in Burkina Faso (4 SMC rounds during the rainy season, maximum ITN coverage, and IPTp), will significantly reduce the incidence of childhood malaria (defined in the following sections) in the intervention arm compared with the control arm (standard-of-care control measures without ivermectin MDA).

#### Secondary Objectives

Secondary objectives include the following:

1. Safety—To measure whether ivermectin MDAs will increase harms for the participants (ie, safety of repeated administrations of high-dose ivermectin)
2. Entomology—To measure entomological indices between the intervention and control arms and over the time of the intervention phase (mosquito survival rate after blood feeding, mosquito population age structure, *Plasmodium* species sporozoite rate, and human exposure to *Anopheles* mosquito bites) between the intervention and control arms
3. Parasitology—To measure the parasitological indices obtained from blood samples collected during the active case surveillance of cohort children between the intervention and control arms and over the time of the intervention phase, including asexual parasite prevalence, species, density, multiplicity of infection, and molecular force of infection (mFOI)
4. Pharmacokinetics and pharmacodynamics—To characterize the pharmacokinetics and pharmacodynamics of ivermectin across the age spectrum and the relationship between ivermectin pharmacokinetics and its entomological effects via membrane feeds

### Study Setting

The study was conducted in villages surrounding the town of Diébougou, situated in the Southwest region of Burkina Faso. The region has highly seasonal intense malaria transmission, typically lasting from June to October or November. SMC was also routinely administered in this region for the 4 months of the rainy season (July to October) to all eligible children aged 3 to 59 months. Village residents represent multiple diverse ethnic Burkinabé groups including Dagara, Mossi, Dioula, and others. Previous entomology studies in the area demonstrated that the primary vector responsible for malaria transmission is *A gambiae sensu strictu* with secondary contributions largely from *A funestus* and *A coluzzii* [18,21,22]. The primary means of subsistence are farming, livestock rearing, and trading.

### Village and Household Selection

In this region, families typically reside in clustered, grouped homes that consist of extended family households. As a first step, a catchment area for potential clusters within the Diébougou health district was determined using Google Earth Pro (version 7.3.6.9345; Google LLC) that was roughly square in shape (approximately 35 km^2^), bounded to the north by the Bougouriba river, to the east by road N12, to the south by the road from Tiankora to Tomena, and to the west by an artificial line that went north from Tomena to the Bougouriba river. Using these satellite images, population centers in this catchment area were outlined based on an analysis of housing structures and an approximation of the number of concessions (extended family households) within each population center. In total, 147 villages or sectors were identified from this analysis, each consisting of between 5 to >100 households. Artisanal gold mining operations were also identified using the maps. From this final map, 18.4% (27/147) of the villages (clusters) were selected based on the following criteria: (1) clusters were accessible to the clinical team during the rainy seasons via motorcycles, which would be their primary mode of transport, providing them with safe road access and allowing them to conduct field visits and return to the field station in Diébougou in 1 day; (2) clusters were at least 2 km away from an artisanal gold mine for the safety of the study team and to minimize contamination from mosquito and human movement to and from these areas; (3) clusters had clearly defined boundaries, allowing field technicians to sample from a single cluster easily without inadvertently including households from neighboring villages; (4) clusters were accessible by study car and no more than 2 km away from an accessible road to facilitate emergency transport of participants to the hospital if necessary; (5) based on an estimated 8 individuals per household, selected clusters had between 20 and 50 households—this was estimated to provide an average of approximately 100 cohort children per cluster, which would enable a single nurse to care for each cluster; and (6) clusters were at least 2 km away from any other enrolled cluster to avoid contamination from mosquito migration. As a final step, in April 2019, the study team selected the final 52% (14/27) of the villages (clusters) based on visits to each village to verify satellite data, household numbers, and village boundaries. All finalized villages and households were enumerated and mapped using handheld GPS devices.

Before the rainy season of year 2, following the review of GPS satellite mapping data, it was determined that a small number of households in a subset of villages were not enrolled in 2019 despite being located at the outer boundaries of participating villages. Owing to their proximity to enrolled households and location within the initial intended catchment area, these households were added to the potential list for enrollment and approached in year 2.

### Study Enrollment and Informed Consent

Before village engagement, approvals were attained from the *Comité d’Ethique Institutionnel pour la Recherche en Santé*, *Comité d’Ethique pour la Recherche en Santé*, and MoH through the *Comité Technique d’Evaluation des Essais Cliniques*, followed by permission from regional health authorities (*Directeur Régional de la Santé* and *Médecin Chef de District*). Following these approvals, community engagement began first with village chiefs and elders and the *Agent de santé Communitaire* (ASC) and subsequently with village residents. All eligible village residents were asked to enroll, which encompassed people of all ages, genders, and reproductive capabilities. Inclusion criteria for enrollment were residence in the study village and ability to understand the information and provide consent or assent. Exclusion criteria for receiving the intervention included (1) sleeping outside of the study village for ≥3 nights per week; (2) height <90 cm; (3) current receipt of SMC; (4) permanent disability or serious medical illness that prevents or impedes study participation and comprehension; (5) pregnancy (screened for in women of childbearing age—12 to 45 years—using a pregnancy urine rapid test the week before each MDA) and older women who have not yet reached menopause; (6) breastfeeding, if the infant is within 1 week of birth; (7) known allergy to ivermectin; (8) possibility of *Loa loa* infection, as assessed by travel history to Angola, Cameroon, Chad, Central African Republic, Congo, Democratic Republic of the Congo, Equatorial Guinea, Ethiopia, Gabon, Nigeria, and Sudan; or (9) enrollment in any other active clinical trials. For the ACD cohort, inclusion criteria were residence in the study village, aged ≤10 years, and parent or guardian consent. Exclusion criteria for the ACD cohort included (1) sleeping outside the study village for ≥3 nights per week or (2) permanent disability or serious medical illness that prevents or impedes study participation.

Eligibility for ACD was determined at the beginning of each intervention season and retained for the remainder of that season:

1. Newborns were eligible for inclusion.
2. Children who turned 11 years during an intervention season remained eligible for the ACD cohort for the remainder of that season.
3. If a participant aged out of the ACD cohort in the first season, they were not eligible for the ACD cohort in the second season.

Eligibility for SMC was determined by the MoH community workers and not by the RIMDAMAL II study team. Participants may age out of SMC (>59 months) in the first season and become eligible for MDA (height ≥90 cm and aged >59 months) in the second season. They did not become eligible for MDA during the season in which this occurred, that is, individuals had to be ≥90 cm at the beginning of the season to receive ivermectin during that season. Cotreatment with ivermectin or placebo and SMC was not allowed during the study because no data were available about the safety of coadministration.

Informed consent was obtained through a multitiered process. After obtaining the permissions stated previously, the head of each household was approached by the study team. Informed consent was obtained from all willing heads of households. Following consent of the head of household, individual consent was obtained from each willing villager. For those aged <18 years, the consent of a parent or guardian was obtained, and for those aged between 12 and 17 years, an additional assent was obtained. Informed consent procedures were repeated before the year-2 intervention season for any potential new villagers who were not present for the informed consent process before the beginning of the study in year 1. Obtaining consent was also an ongoing process throughout the trial, and any participant who refused treatment or wished to withdraw from the study was able to do so without prejudice.

### Cross-sectional Transect Household Selection

A subset of villages and households was selected for more intensive sampling of both humans and mosquitoes to enhance parasitological and entomological secondary studies. Before the beginning of the trial, 6 villages were chosen at random by the study pharmacist, who was unblinded, such that 3 (50%) villages were intervention clusters and 3 (50%) villages were control clusters. Within each of these villages, 8 households were chosen based on GPS mapping and location on spatial transects (east-west and north-south). Households closest to the transects were selected based on location: 2 innermost and 2 outermost households on each transect (8 households per village; 48 households in total). Selected households were subject to more frequent cross-sectional sampling of all individuals living in the household (regardless of age) and to intensive entomological sampling.

### Trial Medication and Interventions

The intervention, ivermectin or ivermectin placebo (Iver P; Laboratorio Elea; 6-mg tablet) at 300 µg/kg dose estimated by height bands, was administered orally for 3 days to eligible individuals who are not receiving SMC. Per the package insert, dosing of ivermectin or placebo was according to height:

1. 90 to 119 cm (15-25 kg)=1 tablet per day for 3 days
2. 120 to 140 cm (26-44 kg)=2 tablets per day for 3 days
3. 141 to 158 cm (45-64 kg)=3 tablets per day for 3 days
4. >158 cm (65-84 kg)=4 tablets per day for 3 days

Ivermectin tablets were stored in a temperature-controlled room at *Institut de Recherche en Sciences de la Santé* in Bobo-Dioulasso until the beginning of each intervention season and then brought to the temperature-controlled field-based pharmacy in Diébougou. Ivermectin or placebo were administered on a monthly schedule from approximately July to October (4 rounds) as a 3-day course to all eligible individuals according to exclusion and inclusion criteria. The intervention period lasted 2 rainy seasons (one in 2019 and another in 2020). The interventional drugs were administered by the study team via the door-to-door delivery method. Whenever possible, dispensing of ivermectin MDA was conducted alongside community health workers distributing SMC to the same households. In the week before each MDA, the study team members requested a urine pregnancy test for any women of childbearing age (12-45 years) or who had not yet reached menopause. For those who were pregnant or did not agree to pregnancy testing, ivermectin was not administered for that round of MDA. Directly observed therapy was conducted for initial doses, whereby the first day of treatment of each month were handled by the study team. If a participant was not available, the dose was left with the village ASC to be provided to the participant later in the day. MDA on the second and third days were also observed, if possible, with doses provided to the ASC for any missing participants. Any doses that were observed and vomited within 30 minutes were readministered. The status of whether doses were observed was recorded for each participant. For all participants who received MDA, active surveillance was conducted on the following day to assess for any adverse events (AEs). Participants were encouraged to consume ivermectin with water and to avoid food for 2 hours after dosing, even though this was not enforced in practice.

IRS was not administered in these villages in the 7 years before the study, aside from a single village that received Actellic 300CS (Syngenta AG) in 2017, and no IRS was deployed during the study. All study villages (14/14, 100%) were part of the periodic MoH ITN distribution cycle, having received pyrethroid ITNs within 2 years before the beginning of the study via mass distribution campaigns. Following the initiation of the study, the MoH relayed that villages in the Diébougou health district were expected to receive new Interceptor G2 ITNs, which were ultimately deployed in October 2019. These new long-lasting insecticidal nets (LLINs) are made with 2 different insecticides (chlorphenapyr and α-cypermethrin) to circumvent the prevalent pyrethroid resistance seen among anophelines in Burkina Faso and much of sub-Saharan Africa.

SMC was provided and distributed by the MoH. It was administered monthly from July to October (for 3 consecutive days) with SP and AQ (eg, SPAQ-CO; Guilin Pharmaceutical). SMC was dosed as 500/25 mg SP and 153 mg AQ for children aged 12 to 59 months and 250/12.5 mg SP and 76.5 mg AQ for children aged 3 to 11 months. As per standard practice in Burkina Faso, the first dose (AQ and SP) is directly observed by MoH staff, and doses on days 2 and 3 (AQ only) are provided to the parents or guardians to administer at home. For pregnant women, IPTp is provided by the MoH and followed World Health Organization policy, which recommended at least 3 treatments, 1 month apart, starting in the second trimester (eg, Fansidar; Roche; 500/25 mg SP per tablet; 3-tablet dosage=1500/75 mg SP).

Ivermectin was also administered to all villages as part of the yearly MDA elimination campaigns, at a single dose of 150 μg/kg orally. According to MoH district leaders, study villages received ivermectin MDA as a single dose for eligible individuals from September 2, 2019, to September 7, 2019, and from November 10, 2020, to November 19, 2020, meaning that study participants received an extra dose of ivermectin 150 μg/kg during the intervention rounds in 2019 and 1 month after the intervention rounds in 2020. Ivermectin MDA was administered by the MoH and not by the study team.

### Outcome Measures

#### Primary Clinical Efficacy Outcome

The primary outcome is the incidence of malaria episodes in the ACD cohort, which comprised children aged ≤10 years, as assessed by active case surveillance by study team nurses assigned to each village and required to visit each child a minimum of once per week during the intervention phases. In this field setting, malaria incidence in children is the most clinically relevant outcome measure for an antivector intervention that targets blood-feeding mosquitoes, and its reduction following repeated ivermectin administration has been validated in our previous RIMDAMAL study [18]. Study nurses were instructed to visit each child weekly to monitor for malaria episodes. At each visit, malaria was defined as a temperature ≥37.5 °C (axillary or equivalent at another site) or history of fever in the last 24 hours with a positive RDT for *Plasmodium* of any species. Study nurses and physicians routinely visited local clinics serving the study villages to capture malaria cases in study participants that were not detected during weekly monitoring in the villages.

#### Secondary Outcomes

Secondary outcomes include the following:

1. Safety and tolerability—AE monitoring occurred throughout the intervention periods in both years through active and passive case surveillance. AE monitoring occurred for all enrolled participants, regardless of age, and will be further characterized by their relationship to the intervention and as serious AEs based on their severity. Active AE monitoring entailed assessments 24 hours following each dose of MDA in all participants available at the time of a nurse’s visit and weekly visits for children in the ACD cohort. Nurses conducted passive AE monitoring over the course of the week during routine study activities.
2. Entomology—Entomological outcomes include the following. (1) *Human biting rate (HBR)*—this is defined as the number of mosquitoes captured per person per period. For aspirated, indoor-resting, blood-fed mosquitoes, HBR is calculated by dividing the house or household catch by the number of humans living in that house or household. For host-seeking mosquitoes caught in light traps placed next to individuals sleeping in tents (outdoors) or under bed nets (indoors), HBR is simply the catch number per person. (2) *Sporozoite rate*—a representative proportion of mosquitoes captured per period and per mosquito species will be tested for the prevalence of infection with *Plasmodium* sporozoites through genetic testing of their heads and thoraces. (3) Changes in participants’ antibody responses to *Anopheles *saliva proteins—paired sets of antibodies from individual participants will be tested for binding to *Anopheles* saliva proteins, and the binding change per person over the study period will be calculated. (4) *The survival of blood-fed mosquitoes*—blood-fed mosquitoes were collected per period, brought back to the field insectary, and held for 3 days to measure their survivorship over that time. A subset of blood-fed mosquitoes from cross-sectional households was stored for the assessment of mosquito biting preference [23].
3. Parasitology—Parasitological outcomes include the following. (1) *Parasite prevalence*—parasite prevalence will be assessed for both *P falciparum*and non-*falciparum* species. (2) multiplicity of infection (also sometimes referred to as complexity of infection) is defined as the number of unique clones per individual at the time of diagnosis of malaria infection. (3) *mFOI* (also sometimes further subclassified as molecular force of blood-stage infection) is defined as the number of new *Plasmodium* clones acquired over time that were not present in the preceding intervals.
4. Pharmacokinetics and pharmacodynamics—Outcomes include (1) the pharmacokinetic profile of ivermectin in 3 different age groups (5-10 years, 11-18 years, and >18 years) sampled over 28 days and (2) the relationship between ivermectin plasma concentration and mosquitocidal effects over time via membrane feeds.

### Power and Sample Size Considerations

**Figure 3:**
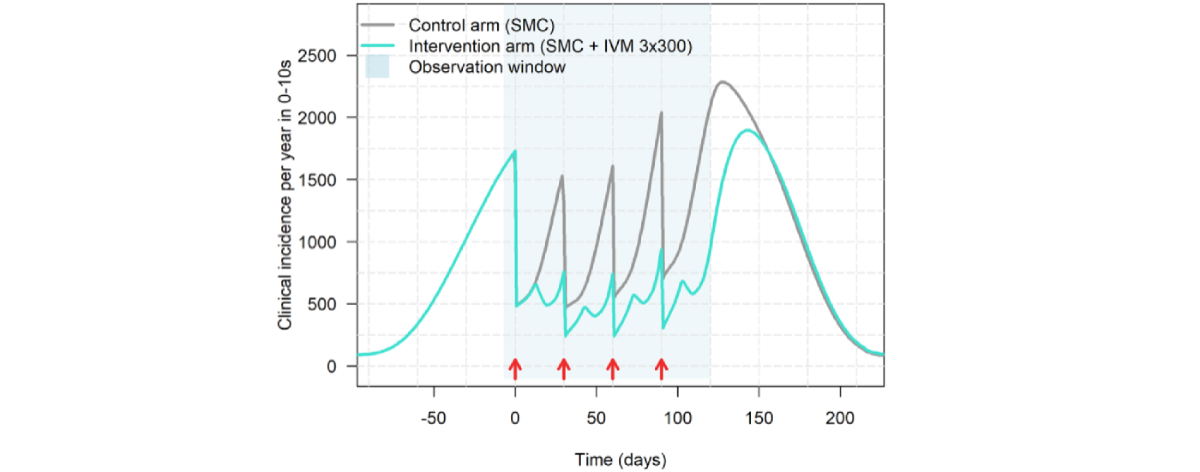
Initial modeled impact of 4 monthly rounds of seasonal malaria chemoprevention (SMC) compared with 4 monthly rounds of SMC and ivermectin (IVM) 3×300. The resulting annualized incidence rates over the observation window were 1.06 in the control arm and 0.60 in the intervention arm.

The primary end point for the trial is the incidence rate of malaria episodes in children aged ≤10 years, as assessed by active case surveillance by study nurses with weekly visits to each child, starting at the first round of MDA in year 1, continuing for 4 months and beginning again with the fifth round of MDA in year 2, and ceasing 4 weeks after the last round of MDA. The dosing regimen, sample size, and power for RIMDAMAL II were guided by the Malaria Transmission model developed at Imperial College, London [24], and are based on data from the RIMDAMAL and Efficacy and Safety of High-Dose Ivermectin for Reducing Malaria Transmission (IVERMAL) trials [1,17-19,25]. The model predicts an effect size in the intervention arm over the control arm of 43% (Figure 3). Calculations assume seasonal incidence rates (per year) of approximately 1.06 and 0.60 in the control and intervention arms, respectively, thus corresponding to an incidence rate ratio of 0.60/1.06=0.57 (ie, incidence rate reduction of 0.43). The initial sample size evaluation shows that RIMDAMAL II has 80% power with 6 villages per arm and 48 children per cluster, assuming a coefficient of variation between clusters of 0.22, which was informed by variation in RIMDAMAL. However, shortly before trial initiation, the sample size was increased from 12 to 14 villages (7 per arm) with approximately 100 children per village or cluster. In a recently published simulation study, the large sample size provides >90% power in the statistical models that were considered for the primary analysis of trial results [26].

This effect size and resulting sample size calculations assumed a moderate level of LLIN coverage with a low-efficacy net owing to high levels of pyrethroid resistance in the region. However, during study planning, it emerged that Interceptor G2 ITNs were to be distributed toward the end of the first year of the intervention. Updating the modeled incidence rates by assuming that the new ITNs would result in an additional 30% reduction, while also increasing the number of children per cluster, meant that even with new LLIN distribution, the number of clusters was still adequate to achieve 80% power.

### Randomization and Blinding Procedures

The randomization unit was the study village (cluster). Before a public randomization event, the study pharmacist, who is the only nonblinded member of the study team, initiated the masking procedure in private, whereby 14 identical cards were made with the numbers 1 to 14 written on them, and these were placed in a container, mixed, and then randomly pulled from the container. In total, 7 numbered cards were randomly placed in each of 2 other containers labeled *ivermectin* or *placebo*. The results of this masking were identically recorded on 3 separate documents, signed by the study pharmacist, and sealed in 3 opaque envelopes kept by the study pharmacist and the 2 principal investigators until the unblinding to be performed after the intervention phase and database cleaning is complete. Following the masking procedure, a public randomization event was conducted before the initiation of the study at the facilities of the district health authority, which was attended by the district medical director, representatives of each village, and members of the study team. At this event, each village’s representative randomly drew 1 card from an opaque container that contained 14 identical cards, except for the numbers 1 to 14 marked on them, and each representative signed and dated a document with the record of the number they drew next to the name of their village. This randomization key was copied for the principal investigators, and the original document was given to the study pharmacist, who used it to match the village to the intervention (ivermectin or placebo) previously assigned to each number during the masking procedure.

The study pharmacist was in charge of the drug stock rooms, and he allocated the tablets for each MDA round according to the original randomization throughout the trial. Drug was distributed in sealed containers labeled with the number code. All study personnel, apart from the pharmacist, remained blinded throughout the study.

### Study Procedures

#### Enrollment

Enrollment forms were completed after obtaining consent from all participants in years 1 and 2 and recorded on study tablets. Year-2 enrollment forms included a reassessment of eligibility criteria and captured additional information about the village participants who were not eligible for MDA owing to them sleeping in the village ≥3 nights per week. Baseline pre-MDA filter paper samples were obtained from a subset of ACD participants in year 1 from each village and from all study participants in year 2.

#### ACD Cohort Follow-up

Active case surveillance was conducted by study nurses visiting each enrolled ACD cohort child weekly following the beginning of the first MDA and ending 30 days following the last MDA of the season. At each visit, the nurse recorded patient information in the electronic case record form, measured the child’s temperature, recorded whether either a history of fever in the past 24 hours or current fever was present, and obtained a blood sample by capillary finger prick method (for RDT, blood smear slide and filter paper [Whatman 3MM] samples were collected). The RDT detected both parasite-derived histidine-rich protein 2 and lactate dehydrogenase (SD Bioline Malaria Ag Pf/Pan; Abbott). All clinical decisions were made based on the RDT result, and if positive for either antigen, results were recorded, and participants were treated with artemether-lumefantrine, according to the Burkina Faso MoH guidelines. Episodes of malaria were classified as complicated based on standard World Health Organization criteria [2]. Patients with complicated malaria were referred to the district hospital immediately. Routine visits were made to local clinics and hospitals to determine whether any participant required management for any AEs that were not captured during routine village-based surveillance.

During each weekly visit, the nurse inquired about any AEs following the receipt of MDA doses or during any intervening time since the last visit. Passive AE monitoring was performed if AEs were communicated to the nurse during other nonstudy visit encounters. Household surveys were performed over the course of the study to ascertain the use of malaria control measures and gather other demographic and social information. In year 2, infection prevention and control measures were implemented to limit the risk of COVID-19 among study staff and participants. Measures included the use of personal protective equipment and the purchase and use of washing stations within each village.

#### AE Monitoring

As noted previously, active and passive AE monitoring occurred in all enrolled villagers, regardless of age. Study nurses and physicians were trained on how to monitor, record, and report AEs. In addition, AEs were followed to resolution when feasible. Severity assessments by the study physicians were performed in consultation with the study nurses and on-site physicians. AEs were graded on a 5-point scale and grouped according to body system and preferred terms using Medical Dictionary for Regulatory Activities. Relationship to the study intervention was determined by the study physician and reviewed by the study’s principal investigators before unblinding.

#### Cross-sectional Transect Household Assessments

Before the beginning of MDA 1 in year 1, cross-sectional households were visited to complete a baseline questionnaire and collect a capillary finger prick sample on filter paper. In year 1, additional blood samples were obtained on filter paper in the middle of the season and approximately 4 weeks following the last MDA. In year 2, filter paper samples were obtained from all cross-sectional household participants before each MDA and 4 weeks following the last MDA. In addition, before and after each season, a hemoglobin measurement was performed using point-of-care HemoCue. Entomology collections in cross-sectional households are described in the following sections. Cross-sectional questionnaires were administered to ascertain the use of malaria control measures and to assess housing and environmental aspects related to malaria risks.

#### Parasitological Assessments

Parasite prevalence and speciation will be determined using molecular assays on DNA extracted from filter paper samples using established methods. *P falciparum* clonality (complexity of infection and mFOI) will be assessed using either length-polymorphic markers or single nucleotide polymorphism–based genotyping using deep amplicon sequencing [27,28].

#### Entomological Assessments

Mosquito sampling was conducted in a subset of cross-sectional villages, within the cross-sectional transect households only. Overall, 2 sampling methods were used; the first method was indoor aspirations of resting mosquitoes collected in the morning in all cross-sectional household sleeping rooms, and the second was light traps placed in 1 cross-sectional household located in the center of the village to collect host-seeking anophelines overnight. For the light traps, one was placed outdoors hanging under the rain tarp of a camping tent that was occupied by a sleeping person, and another was placed indoors hanging next to a person sleeping under a bed net. Mosquito sampling intervals occurred in these 6 cross-sectional villages over the course of a week and were conducted 1 week after each MDA and 3 weeks after each MDA for a total of 16 sampling intervals across the entire intervention period.

#### Pharmacokinetics and Pharmacodynamics Assessments

In October 2020, following the third round of ivermectin MDA, a pharmacokinetics and pharmacodynamics substudy was performed for individuals residing in 6 villages (n=3, 50% intervention and n=3, 50% control villages). In total, 178 participants were enrolled in the substudy, approximately 90 each from the intervention and control villages, with all team members except the pharmacist blinded to the village study arm. Participants were enrolled to achieve roughly equal numbers from three age groups: (1) 5 to 10 years, (2) 11 to 18 years, and (3) >18 years. Sparse pharmacokinetics sampling was conducted for each individual at up to 7 time points over 28 days, with additional blood collected for entomological and safety assessments (chemistries and blood counts). Pharmacokinetics data will be analyzed using nonlinear mixed effects modeling, and entomological assessments will be performed using membrane feeds on participant plasma.

### Data Management

Data were gathered in an electronic data capture system (REDCap [Research Electronic Data Capture; Vanderbilt University] database system). All electronic case report forms containing participant demographic and clinical data (active case surveillance data on the children cohort and AE data for all participants) were entered on password-protected tablets that were maintained and updated daily by the clinical team working at the field site. Only the study team will have access to the source data. Long-term maintenance of the participant codes is held only by the study’s principal investigators on password-protected computers and files. Database records were individually checked for inconsistencies and cross-checked with the study team and other data sources, as applicable. Study data devoid of identifiers will be provided to the research community following the publication of the primary outcomes.

### Ethics Approval and Regulatory Oversight

Institutional review board approvals were obtained and maintained from the Colorado State University (19-9144H/1691), Yale Human Investigations Committee (under a Streamlined, Multisite, Accelerated Resources for Trials Institutional Review Board Reliance platform agreement with the Colorado State University; 2000024138), *Comité D’Ethique Institutionnel Pour La Recherche en Sciences de La Santé* (A031-2018), *Comité D’Ethique Pour La Recherche en Santé* (2019-01-009), and *Comité Technique D’Examen Des Demandes D’Autorisation D’Essais Cliniques* (2019-0580). The protocol was approved by the National Institutes of Health Division of Microbiology and Infectious Diseases (protocol number 18-0007). An independent data and safety monitoring board was convened by the Division of Microbiology and Infectious Diseases. Protocol violations and serious AEs were reported to the abovementioned regulatory bodies according to institutional requirements. An independent study monitor (an in-country contract research organization, CliMAfrica-CRO, Burkina Faso) provided external monitoring for the study, beginning before study initiation and until completion of the study. The CONSORT (Consolidated Standards of Reporting Trials) extension for cluster trials will be completed and made available at the time of reporting primary study results for publication.

### Statistical Analysis

#### Primary Clinical Outcome

We will use an intention-to-treat analysis method for our primary clinical outcome, which is the incidence rate in children in the ACD cohort [26]. Person time will be defined in 7-day consecutive intervals, with ≥1 visits occurring during each interval considered as a single eligible person week. A Poisson mixed effects regression model with a village-level random effect will use the counts of malaria episodes with exposure time (modeled as an offset) in each participant to estimate the malaria incidence rate for all participants in the intervention and control villages. The primary analysis will be adjusted for other covariates such as ITN use and participant age. The estimate for the primary analysis is the exponentiated coefficient for the treatment covariate across both seasons. This rate ratio is the ratio of malaria incidence rate in the treatment villages to the rate in the control villages. The model will be fit with a Kenward Roger *df* correction to account for the limited number of clusters. The primary conclusion about the relative efficacy of ivermectin MDA will be based on the treatment effect coefficient, its 95% CI, and *P* value testing difference from 0 (rate ratio of 1). The primary analysis method and model were selected based on the results of a simulation study of the statistical properties of many models that have been proposed for the analysis of cluster-randomized trials using count-based outcomes [26].

The primary analysis will evaluate the efficacy of ivermectin treatment as described previously. The robustness of this conclusion will be evaluated through analyses that will adjust for other covariates and assess the strength of the rate ratio in key subgroups. Sensitivity analysis will be performed to assess the impact of censoring a participant’s time at risk for 14 days following the diagnosis and treatment of malaria owing to the impacts of treatment on risk of recurrent malaria.

Subgroup analyses will be performed to help inform the mechanism of action of ivermectin and determine whether the effects are similar across important subgroups. Subgroups include (1) children aged 0 to 3 months, who receive neither SMC nor intervention; (2) children aged 3 to 59 months, who receive SMC only; (3) children aged >59 months but are <90 cm in height, and therefore do not receive SMC or the intervention; and (4) those who receive MDA only (height ≥90 cm and aged ≤10 years).

#### Safety Analysis

*Safety analysis* will be assessed by relative risk (RR) in the following groups: (1) all participants were followed actively and passively for AEs, and the risk ratios of AEs of those in the intervention versus control clusters will be compared; (2) ACD-cohort SMC-treated children only—RR of AEs in children who receive SMC and live in ivermectin-treated clusters versus those who receive SMC and live in placebo-treated clusters; (3) ACD-cohort ivermectin-treated or placebo-treated children only—RR of AEs in those children receiving ivermectin MDA versus those receiving placebo; and (4) non-ACD participants who were enrolled in the study were followed actively for 24 hours after each study intervention and then passively for the rest of the MDA round.

## Results

Consent process and recruitment for the study began on July 13, 2019. The first round of MDA began on July 26, 2019, and the last round of MDA in year 1 began on October 16, 2019. MDA in year 2 began on July 20, 2020, and the last round of MDA began on October 12, 2020. SMC and ivermectin MDA distribution occurred on the same days for all MDA except MDA 1 in 2019, in which ivermectin MDA was delayed relative to SMC by 2 days. Statistical analysis will be conducted once the database is completed, cleaned, and locked, and the clinical trial protocol is publicly available. The study team will be unblinded after the clinical trial protocol and statistical plan is finalized and publicly available.

## Discussion

### Principal Findings

With gains in malaria stagnating since 2015 and reversals being seen in several countries, particularly in sub-Saharan Africa, novel control methods are urgently needed [3]. Novel interventions in conjunction with other measures will likely be needed, as no single intervention is expected to have a sufficient, durable, and sustainable impact to reduce malaria morbidity and mortality in the long term. Previous studies by our group and others demonstrated that ivermectin, when used at 150 μg/kg, similar to doses used for MDA campaigns to eliminate lymphatic filariasis, onchocerciasis, and scabies (150-400 μg/kg), showed lethal and sublethal effects on mosquitoes following blood meal ingestion [15,29-32]. The same dosage was then shown to affect malaria incidence when administered more frequently (every 3 weeks) in the RIMDAMAL trial [18]. Modeling studies have demonstrated that a monthly regimen of 300 μg/kg administered on 3 consecutive days is likely to have a significant impact on malaria incidence [1]. The safety and mosquitocidal impact of this regimen were recently demonstrated in a randomized trial of adults with malaria living in Kenya [17].

RIMDAMAL II builds on these insights and will assess whether the high dose of ivermectin MDA, when administered in operational conjunction with SMC delivery, is associated with significant reduction in malaria incidence in children aged <10 years, as compared with standard interventions alone (SMC and ITN use). Malaria incidence was chosen as the primary outcome, as this was felt to reflect a meaningful outcome measure that could have an impact on policy decisions. The study is cluster randomized and placebo controlled, with all study investigators being blinded to intervention allocation, which is a rigorous design to test the community-level impact of this novel intervention. The study intervention was also conducted over 2 seasons and involved both active and passive follow-up of AEs for all enrolled participants, thus providing a strong evidence base to assess the safety of repeated high-dose ivermectin for malaria transmission reduction. Secondary outcomes will provide parasitological, entomological, and pharmacokinetic outcome measures to further enhance our understanding of the mechanisms by which ivermectin may exert its effects. Additional sampling in the year following the intervention will provide information on any postintervention effects of ivermectin MDA.

Community-level trials are subject to several challenges during planning and execution. The identification of appropriate clusters that are sufficiently distanced from one another to avoid contamination and of appropriate size to enable rigorous surveillance of all participants can be challenging, but it also reflects the reality of spatial demographics in rural African settings. During the planning stages, attempts were made to identify ideal villages for inclusion, while balancing with practical limitations in the field (refer to previous sections).

Cluster-randomized, community-based trials are also open to external factors that can challenge the execution phases of the study. During RIMDAMAL II, the COVID-19 pandemic spread around the globe just before the beginning of the second intervention season. In recognition of the potential impact that the COVID-19 pandemic could have on multiple aspects of the study (safety of the staff and participants, availability of supplies, ability to differentiate malaria and COVID-19, etc), extensive discussions and preparations occurred before year 2 of the study. Efforts included the training of the staff on appropriate infection prevention and control measures, purchase and shipment of personal protective equipment from the United States to Burkina Faso, and purchase of handwashing stations for each village. At the conclusion of year 2 of the intervention, there was no overt evidence of COVID-19–related clinical impacts in our enrolled villages; however, precise data on COVID-19 case counts in the region were not available.

A second challenge was the decision by the Burkina Faso MoH and certain research partners to deploy ITNs with different insecticide chemistries in Southwest Burkina Faso. In the region of Diébougou, new Interceptor G2 ITNs were deployed in October 2019, at the end of year-1 intervention season. The timing of distribution created challenges and opportunities, which will be assessed in the analysis, as both year-1 and year-2 outcomes largely occurred under the use of deltamethrin and chlorphenapyr or α-cypermethrin nets.

RIMDAMAL II will be the first cluster-randomized trial to directly assess the impact of repeated high-dose ivermectin MDA on malaria incidence. The trial uses high-dose, monthly ivermectin coupled operationally with SMC, and the assessment of primary outcome includes children who received only SMC (those aged 3-59 months) and those who received only ivermectin (and no SMC; those aged 60 months to 10 years). Statistical analysis planning supports the adequacy of the cluster size and the approach to assess the primary outcome [26]. The study encountered challenges with the emergence of COVID-19 globally and the deployment of ITNs with new chemistries locally in year 2. Following the final analysis and dissemination of results, if the intervention is found to be efficacious and safe, the design will facilitate the potential consideration of this multilayered novel approach for malaria control in seasonal high-transmission settings.

### Conclusions

RIMDAMAL II will be the first cluster-randomized trial to directly assess the impact of repeated high-dose ivermectin MDA on malaria incidence in children. The results will inform policies and future studies on the use of mosquitocidal agents as novel components for malaria control.

## Abbreviations

ACD: active case detection
AE: adverse event
AQ: amodiaquine
ASC: Agent de santé Communitaire
CONSORT: Consolidated Standards of Reporting Trials
HBR: human biting rate
IPTp: intermittent preventive treatment in pregnancy
IRS: indoor residual spraying
ITN: insecticide-treated bed net
IVERMAL: Efficacy and Safety of High-Dose Ivermectin for Reducing Malaria Transmission
LLIN: long-lasting insecticidal net
MDA: mass drug administration
mFOI: molecular force of infection
MoH: Ministry of Health
RDT: rapid diagnostic test
REDCap: Research Electronic Data Capture
RIMDAMAL: Repeat Ivermectin Mass Drug Administration for Malaria Control
RR: relative risk
SMC: seasonal malaria chemoprevention
SP: sulfadoxine-pyrimethamine

## Data Availability

Data for the trial will be made available on the Clinical Epidemiology Database Resources [33].
